# 1DCNN-BiLSTM-transformer hypertension risk prediction model based on APW

**DOI:** 10.3389/fmicb.2025.1714654

**Published:** 2025-11-11

**Authors:** Yan Peng, Lu Ma, Huiyu Zhou, Jiao Li, Jie Wang

**Affiliations:** 1 School of Management, Capital Normal University, Beijing, China; 2 School of Computing and Mathematical Sciences, University of Leicester, Leicester, United Kingdom; 3 Institute of Medical Information, Chinese Academy of Medical Sciences/Peking Union Medical College, Beijing, China

**Keywords:** arterial pressure waveform, hypertension, gastrointestinal microbiome, CNN, RNN, transformer

## Abstract

**Introduction:**

Hypertension has a multifactorial etiology. Recent studies have revealed a link between hypertension and gut microbiota dysbiosis. Pulse wave analysis holds significant clinical value for hypertension risk assessment. While research on deep learning models utilizing photoplethysmography (PPG) for hypertension classification has advanced, limitations persist. PPG offers limited richness and accuracy for characterizing blood pressure-related pathological information. In contrast, Arterial Pressure Waveform (APW) provides richer pathological information and exhibit stronger correlations with clinically interpretable features. However, deep learning research using APW for hypertension classification remains limited, as existing studies focus primarily on local feature extraction and neglect global temporal dynamics.

**Methods:**

To address these challenges, we propose a novel 1D-CNN-BiLSTM-Transformer architecture for hypertension risk assessment based on APW, where the 1D-CNN module extracts waveform morphology features from signals within individual pressure segments, the BiLSTM module models long-range temporal dependencies from signals within each segment, and the Transformer module explicitly captures nonlinear interaction from signals across different pressure segments through multi-head self-attention mechanisms.

**Results:**

We use the multi-channel APW database from the Population Health Data Archive (PHDA), containing hypertensive and non-hypertensive cases with APW signals acquired from six traditional Chinese medicine points (left-cun, left-guan, left-chi, right-cun, right-guan, and right-chi) to evaluate the model’s performance. The model outperforms the current state-of-the-art methods in accuracy, precision, recall, and F1 score across all six points.

**Conclusion:**

The proposed model enhances classification performance. The physiologically driven interpretable analysis demonstrates that APW can reflect pathophysiological features associated with gut microbiota dysbiosis. The model-driven interpretable analysis offers a decision-making basis for clinical diagnosis.

## 1 Introduction

The development of hypertension results from the interplay between genetic and environmental factors (Townsend et al., 2016). To date, genetic contributors to hypertension have been extensively studied. Studies have suggested that dysbiosis of the gut microbiota is closely associated with the progression of hypertension. Alterations in the abundance of certain gut microbial strains may suppress or attenuate immune responses related to chronic inflammation, indicating their potential role as biomarkers for the prevention and treatment of hypertension (Yu et al., 2024). Therefore, exploring novel therapeutic targets from the perspective of the gut microbiome etiology is both highly feasible and necessary.

Blood pressure (BP) is defined as the lateral pressure exerted by blood flow per unit area on blood vessel walls. It is categorized by vessel type: capillary pressure, venous pressure, and arterial blood pressure (ABP), with ABP being the most commonly referenced measure (Polska et al., 2007). Extensive research demonstrates a strong correlation between pulse wave signals and ABP. For instance, Luo et al. (2006) invasively measure ABP in the canine aorta while simultaneously capturing non-invasive pulse waves, observing consistent waveform morphology and temporal relationships. Martinez et al. (2018) further quantify this similarity in both the time and frequency domains, reporting an average Pearson correlation coefficient exceeding 0.9. Complementing these findings, Abhay et al. (2017) analyze specific feature-based similarities, finding high Pearson correlation coefficients for amplitudes (0.822), normalized time periods (0.99), and normalized rise times (0.78) between ABP and pulse wave signals. Collectively, this evidence strongly suggests that pulse wave signals provide valuable information for assessing BP.

Photoplethysmography (PPG) and Arterial Pressure Waveform (APW) represent two distinct pulse wave types acquired through different sensing modalities. PPG signals, obtained using optical sensors, detect blood volume fluctuations caused by light absorption or reflection within the microvasculature (small arterioles, small venules, and capillaries) in the subcutaneous microcirculation (Lee et al., 2021). There has been considerable advancement in BP prediction studies based on PPG (De Nisio et al., 2025; Shoaib et al., 2025; Shimazaki et al., 2019). APW signals are captured using pressure sensors directly coupled to the brachial or radial artery, thereby reflecting the dynamic pressure characteristics of the arterial wall (Avolio et al., 2009). Due to inherent limitations in its acquisition method, PPG signals are more constrained in characterizing the richness and fidelity of BP-related pathological information compared to APW signals. Furthermore, when deep learning networks process APW signals for classification, the primary classification features exhibit more direct and robust correlations with clinically interpretable physiological indicators. Consequently, APW signals are better suited as input for deep learning models targeting hypertension classification, offering enhanced potential for both model performance and interpretability.

Deep learning has gained significant momentum in recent research for pulse wave-based BP prediction (Nuryani et al., 2023). Among prevalent architectures, Convolutional Neural Networks (CNNs) are widely adopted, leveraging their powerful feature extraction capabilities to achieve commendable performance on this task (Khodabakhshi et al., 2022; Schlesinger et al., 2020; Qin et al., 2021; Yan et al., 2019; Liu, 2021). However, CNNs possess inherent limitations: they can only extract local features due to their restricted receptive fields, and they are not specially designed for processing one-dimensional (1D) sequential data, whereas the pulse wave data represents quintessential 1D time series (Tian et al., 2025). To address these constraints, Recurrent Neural Networks (RNNs) have been proposed to model the temporal dependencies within pulse wave data (Aguirre et al., 2021; El-Hajj and Kyriacou, 2021a; El-Hajj and Kyriacou, 2021b; Liu et al., 2024). Nevertheless, standard RNNs often struggle to capture very long-range dependencies effectively (Zhang et al., 2023). Further innovations involve hybrid architectures that integrate CNN and RNN components to leverage their complementary strengths in local feature extraction and temporal dependency modeling, respectively (Hung et al., 2025; Wang et al., 2020; Panwar et al., 2020; Zhang et al., 2024; Leitner et al., 2022). Recently, the Transformer architecture (Vaswani et al., 2017) has garnered extensive attention across diverse domains. Its core self-attention mechanism excels at recognizing long-range relationships within sequences and learning global contextual features, making the Transformer inherently more suitable for modeling the complex temporal dynamics of pulse wave signals compared to both CNNs and traditional RNNs.

To address the limitations of existing approaches for pulse wave-based hypertension classification, we propose a novel 1DCNN-BiLSTM-Transformer architecture based on APW analysis (Ju et al., 2024; Peng et al., 2024a; Peng et al., 2024b; Feng et al., 2023). This model integrates complementary strengths: the 1DCNN-BiLSTM module extracts the local features from signals within individual pressure segments, while the Transformer module captures the complex, long-range interdependence among signals across different pressure segments. The feature fusion module combines these hierarchically learned representations for final classification. We evaluate model performance using the multi-channel APW database containing hypertensive and non-hypertensive cases from the Population Health Data Archive (PHDA) (Geng, 2024). The dataset includes APW data from 495 hypertensive and 611 non-hypertensive subjects, collected at three points on each hand (referred to as cun, guan, and chi in Chinese medicine) under 14 step-pressure gradients ranging from 10 to 140 mmHg at 10 mmHg intervals. Validation experiments demonstrate that our proposed model significantly outperforms state-of-the-art typical deep learning methods for pulse wave-based hypertension classification in accuracy, precision, recall, and F1 score across all six collection points. Furthermore, to bridge the gap between algorithmic output and clinical utility, we conduct the interpretability analysis from three perspectives: (1) model performance variation analysis under six collection points, (2) attention weight analysis of signals for each pressure segment, and (3) spatial feature importance analysis using Grad-CAM (Selvaraju et al., 2020). The synergistic combination of superior predictive performance and clinically grounded interpretability establishes a robust methodological framework for hypertension risk assessment.

## 2 Materials and methods

### 2.1 Network architecture

**Figure 1:**
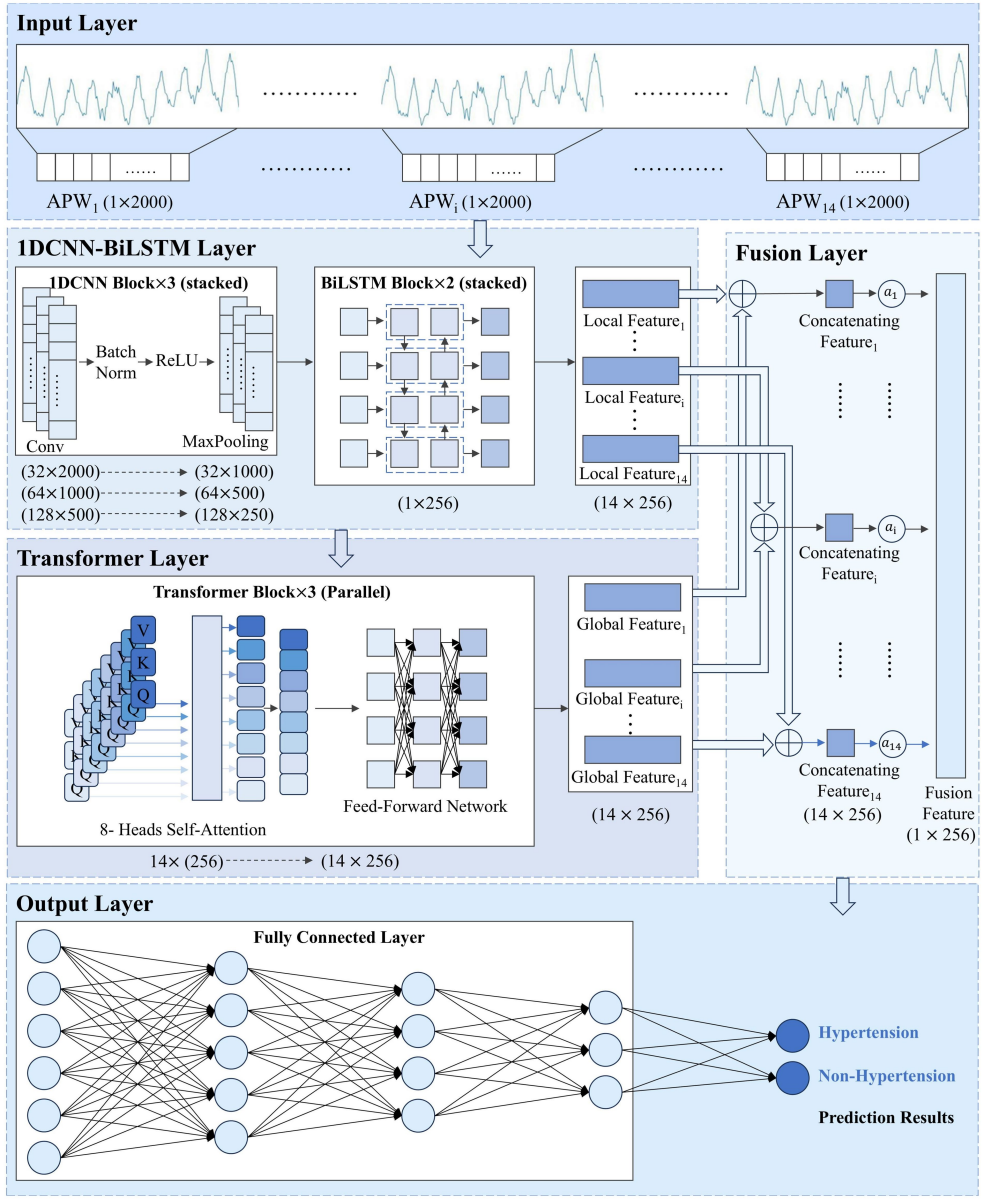
1DCNN-BiLSTM-Transformer model structure. $\mathrm{AP}W_{i}$ represents the pulse wave sequence under the i-th pressure level. $\text{Local Featur}e_{i}$ , $\text{Global Featur}e_{i}$ , and $\text{Concatenating Featur}e_{i}$ denote the local features, global features, and concatenated features, respectively, extracted from the pulse wave sequence at the i-th pressure level. $\alpha_{i}$ refers to the attention weight assigned to the i-th pressure level. Fusion Feature represents the final fused feature.

The overall architecture of the 1DCNN-BiLSTM-Transformer model we proposed is shown in Figure 1.

### 2.2 1DCNN-BiLSTM module

The One-Dimensional Convolutional Neural Network-Bidirectional Long Short-Term Memory (1DCNN-BiLSTM) module extracts local features from signals within each pressure segment. The 1DCNN module extracts morphological features, and the BiLSTM module captures temporal dependencies. The 1DCNN-BiLSTM module preserves pressure gradient information and signal features within each pressure segment.

#### 2.2.1 1DCNN module

The convolutional layer identifies specific waveform fragments through convolutional kernels, while its translation invariance ensures that these kernels can detect the waveform features at each position. The 1DCNN module comprises three convolutional blocks. These three convolutional blocks employ convolutional kernels of decreasing sizes to extract waveform features at different scales: The first layer captures basic waveform features such as peaks and valleys; the middle layer combines first-layer features to identify more complex waveforms such as main waveforms, pre-prestroke waveforms, and re-prestroke waveforms; and the final layer further integrates these features to capture local morphological details. The one-dimensional convolution operation for signal feature extraction is shown in Equation 1:


$$y[i]=\sum_{j=0}^{k=1}w[j]\cdot x[i+j]$$ (1)

x is the APW sequence within each pressure segment, w[j] is the weight of the j th element in the convolution kernel, x[i+j] is the element taken from x that corresponds with the current convolution kernel, and y[i] is the output feature value at position i .

#### 2.2.2 BiLSTM module

With 2000 time steps per pressure segment, APW sequences are so lengthy that the 1DCNN module alone cannot capture global features within each pressure segment. Therefore, we introduce the BiLSTM module after the 1DCNN module to extract long-range temporal dependencies. For pulse wave signals, both forward evolution information from the systolic to diastolic phase and backward information reflected back from the distal end are equally significant for hypertension diagnosis. Consequently, we configure the LSTM module as bidirectional, enabling simultaneous forward and backward sequence processing to capture bidirectional signal dependencies.

### 2.3 Transformer module

The Transformer module captures interdependencies among signals across different pressure segments, learning pressure change patterns of the APW signals. The module comprises three transformer blocks connected in parallel, each containing an 8-head self-attention layer and a feed-forward network. The multi-head attention layer is designed to enable the model to consider all other positions in the sequence when processing each position, thereby capturing long-range intra-sequence dependencies. Feature importance is dynamically adjusted through the mechanism to enhance signal feature representations within each pressure segment according to global forward and backward information.

Each block accepts the local feature sequence $L=(\text{Local Featur}e_{1},\ldots .,\text{Local Featur}e_{i},\ldots ,\text{Local Featur}e_{14})$ extracted by the 1DCNN-BiLSTM module as input. Within each block, the computation of Query (Q), Key (K), and Value (V) matrices, followed by the scaled dot-product attention mechanism, enables interactive modeling of the input, allowing information at each position to directly attend to and integrate features from all other positions. The feature sequences $\left\{O^{\left(1\right)},O^{\left(2\right)},O^{\left(3\right)}\right\}$ output by the three blocks are adaptively integrated through a gated fusion, which computes dynamic gating weights (Equations 2 and 3). The global feature sequence $G=(\text{Global Featur}e_{1},\ldots ,\text{Global Featur}e_{i},\ldots ,\text{Global Featur}e_{14})$ is ultimately produced via weighted summation (Equation 4). The sequence G comprehensively encapsulates information from all pressure segments.


$${\tilde{O}}^{\left(k\right)}=\text{Tanh}(O^{\left(k\right)}W_{o}^{\left(k\right)}+b_{o}^{\left(k\right)})$$ (2)

$$g(k)=\text{softmax}({\tilde{O}}^{\left(k\right)}W_{g}+b_{g})$$ (3)

$$G=\sum_{k=1}^{3}g_{k}⊙{\tilde{O}}^{\left(k\right)}$$ (4)

${\tilde{O}}^{\left(k\right)}$ is the transformed feature sequence of the k-th Transformer block, with the transformation serving to align the outputs of the three blocks into a unified feature space. g(k) is the dynamic gating weight vector associated with the k-th block.

### 2.4 Feature fusion module

The feature fusion module fuses local features from signals at each pressure segment (extracted by the 1DCNN-BiLSTM module) with global features from the entire signal (extracted by the Transformer module) in the feature dimension.

For the i th pressure segment, the concatenating feature $C_{i}$ calculation process is shown in Equation 5:


$$C_{i}=\text{ReLU}(W_{c}\cdot [L_{i}⊕G_{i}]+b_{c})$$ (5)

$C_{i}$ s are input into an attention pooling layer to learn their importance weights $\alpha_{i}$ s (Equation 6), and then $C_{i}$ s are weighted and fused to generate a single feature vector (Equation 7), $F_{\text{fused}}$ that comprehensively represents APW signal information within all pressure segments.


$$\alpha_{i}=\frac{\exp(W_{\alpha }\cdot C_{i}+b_{\alpha })}{\sum_{i=1}^{14}\exp(W_{\alpha }\cdot C_{i}+b_{\alpha })}$$ (6)

$$F_{\text{fused}}=\sum_{i=1}^{14}\alpha_{i}\cdot C_{i}$$ (7)

Finally, $F_{\text{fused}}$ is fed into the classification module, which consists of three fully-connected layers that perform nonlinear mapping and integration of the fused high-dimensional features. The network ultimately produces a binary prediction indicating either “Hypertension” or “Non-Hypertension,” achieving the automated classification of pulse wave sequence data.

## 3 Experiments

### 3.1 Dataset

The data are obtained from the APW dataset shared on the Population Health Data Archive (PHDA).

**Figure 2:**
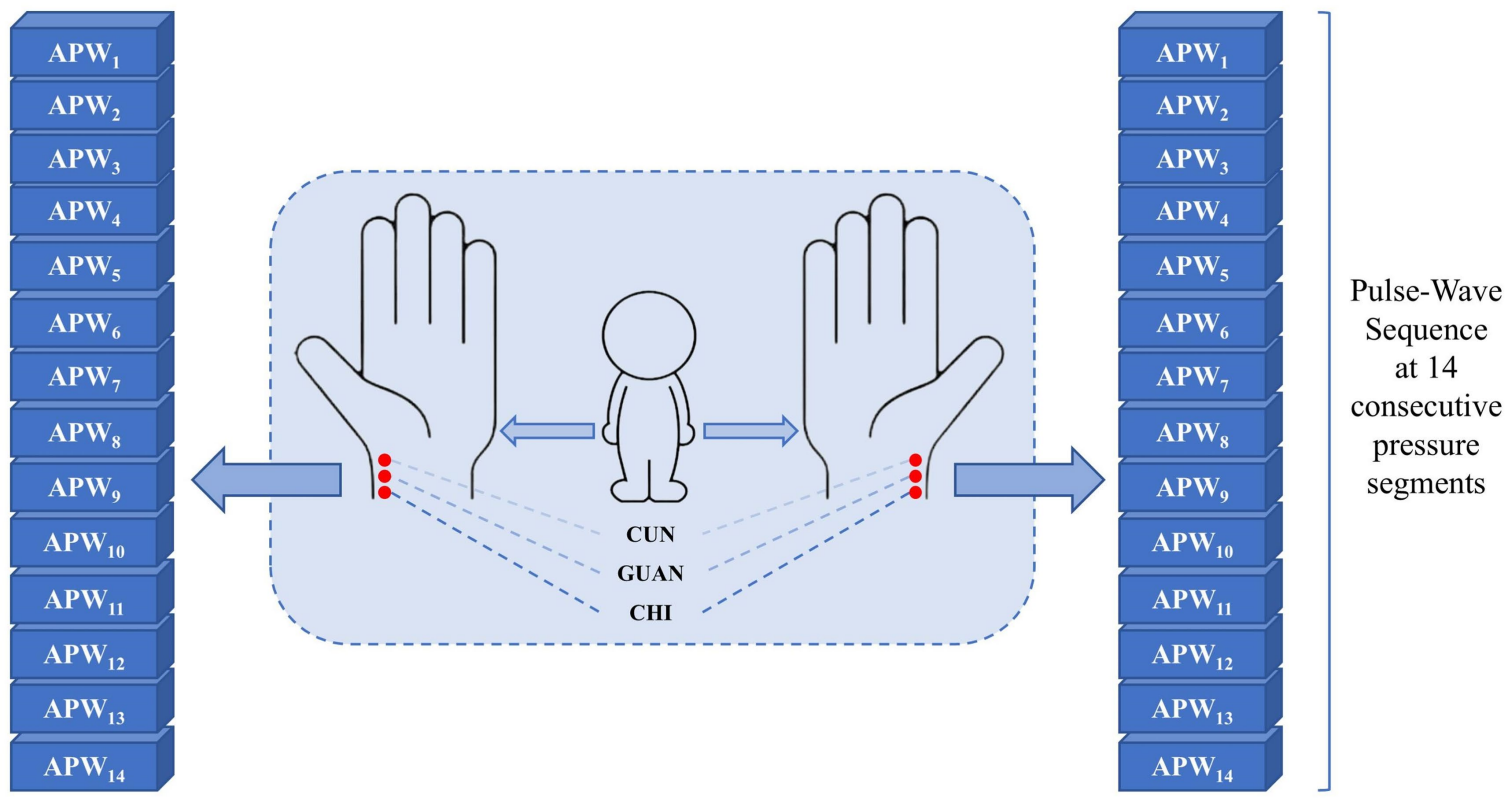
APW signal corresponds to six points (left-cun, left-guan, left-chi, right-cun, right-guan, and right-chi) under 14 step-pressure gradients ranging from 10 to 140 mmHg at 10 mmHg intervals. The Cun, Guan, and Chi are three distinct TCM points on the medial wrist. The APW signal obtained from each point can, respectively, indicate the functional status of different Zang-fu organs.

Data collection employed a multi-channel pulse acquisition instrument consisting of an airbag pressurization device and three composite pressure sensors. The acquisition process operates as follows: the airbag pressurization device provides step pressures from 10 to 140 mmHg in 10 mmHg intervals, and the cuff is inflated to generate the specified pressure for approximately 10 s with a 225 Hz sampling frequency at each pressure step, while APW signals and static pressure information from the three sensors are recorded in real time until the next inflation command. In this way, 14-order static pressure values and corresponding APW sequence data from three reference points on each hand under 14 pressure gradients from every subject (495 with hypertension and 611 without hypertension) are collected. These three points are designated as cun, guan, and chi in traditional Chinese medicine, reflecting human body health status across different dimensions. The collection points and results are shown in Figure 2, with dataset details presented in Table 1.

**Table 1:** The details of the dataset.

Labels	Sample size	Points	Pressure segments (mmHg)
Hypertension	495	Left-Cun, Left-Guan, Left-Chi, Right-Cun, Right-Guan, Right-Chi	10, 20, 30, 40, 50, 60, 70, 80, 90, 100, 110, 120, 130, 140
Non-Hypertension	611		

### 3.2 Data preprocessing

For the APW sequence data at each acquisition point, data preprocessing is performed in three aspects: conventional data cleaning, noise reduction, and baseline drift removal.

#### 3.2.1 Conventional data cleaning

The collected data for each pressure segment contains approximately 2,250 points. During data collection, APW signals briefly drift during each pressurization process. Furthermore, neural networks require equal points in each pressure segment. Therefore, if n points exist under each pressure section, we remove points a from the starting point and b before the ending point during each pressurization process to ensure the length under each pressure segment is 2000 (Equations 8 and 9).


a=⌈(n−length)/2⌉ (8)

b=⌊(n−length)/2⌋ (9)

#### 3.2.2 Noise reduction

During actual collection, high-frequency noise becomes mixed into APW signals due to internal instrument noise, electromagnetic interference, motion artifacts, and other interferences, so low-pass filtering technology is needed to reduce the high-frequency noise component in collected signals. Wavelet threshold denoising (Zhang et al., 2016) can eliminate the high-frequency noise influence on signals and extract the main features.

We represent the collected signal as f(t)=s(t)+h(t) , where f(t) is the unprocessed noisy signal, s(t) is the useful signal, and h(t) is the high-frequency noise signal. The signal after wavelet threshold denoising is g(t) . Based on extensive experimental comparisons, the coif6 wavelet is selected as the wavelet basis, with a decomposition level of 3. The threshold is determined using the fixed threshold estimation, and the high-frequency coefficients are processed with the soft thresholding function. The entire wavelet threshold denoising process can be represented by the following pseudo-code:Begin:Input:f(t)
// Initialization
Choose wavelet basis as ‘coif6’
Set decomposition level to 3
Select fixed threshold estimation method
Select soft threshold function
// Wavelet decomposition
(LowFreqCoeffs, HighFreqCoeffs)=WaveletDecompose(f(t), ‘coif6’,3)
// Threshold determination
Threshold=FixedThresholdEstimation(HighFreqCoeffs)
// High-frequency coefficient processing
for each level in HighFreqCoeffs:
   for each Coeff in HighFreqCoeffs(level):
      if abs(Coeff)>Threshold:
        ProcessedHighFreqCoeffs(Level).append(Coeff)
      else:
        ProcessedCoeff=SoftThresholdFunction(Coeff,Threshold)
        ProcessedHighFredCoeffs(level).apped(ProcessedCoeff)
// Wavelet reconstruction
g(t) = WaveletReconstruct(LowFreqCoeffs, ProcessedHighFreqCoeffs, ‘coif6’, 3)
Output:g(t)
End


#### 3.2.3 Baseline drift removal

During actual collection, low-frequency noise becomes mixed into APW signals due to subject breathing or slight body movement, temperature drift, voltage drift, and other disturbances, causing APW signals to deviate from their baseline and present slow, non-periodic fluctuations. Empirical Mode Decomposition (EMD) (Kopsinis and McLaughlin, 2009) adaptively decomposes the signal layer by layer from high frequency to low frequency into multiple Intrinsic Mode Functions (IMFs) according to its characteristics. The low-frequency noise is eliminated by removing IMF1, while the high-frequency IMFs are retained and reconstructed as the signal after removing the baseline drift.

**Figure 3:**
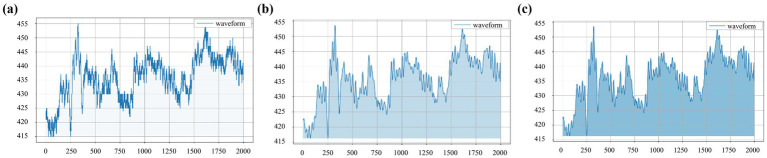
Waveforms obtained from the left-cun pulse of one subject under the 10 mmHg pressure: **(a)** original signal, **(b)** signal after wavelet threshold denoising, and **(c)** signal after baseline drift removal.

After data preprocessing, the waveform becomes smoother and retains the waveform features of the signal to the greatest extent. As shown in Figure 3, the waveforms of original and preprocessed signals remain consistent, establishing a solid foundation for subsequent analysis.

### 3.3 Data augmentation

For the small datasets in this study (495 hypertensive samples and 611 non-hypertensive samples), we adopt data augmentation to introduce random variations of the original data to reasonably and effectively expand the scale and diversity of the training set, which enables the model to learn more robust and stable feature representation while reducing overfitting and enhancing model generalization ability.

Considering APW characteristics, we design the following three data augmentation methods to expand the training set while preserving physiological fidelity:

1. Injection of band-limited Gaussian noise: While preprocessing typically removes high-frequency noise, APW signals may exhibit subtle, low-amplitude stochastic variations due to physiological micro-tremors or sensor limitations. To simulate this realistically without reintroducing discarded noise, we inject low-intensity, band-limited Gaussian noise with constrained probability.
2. Random cyclic time shifting: To account for inherent physiological fluctuations in the periodicity of APW signals, we simulate this natural phase variability by applying random cyclic shifts along the time axis.
3. Random amplitude scaling: To simulate natural amplitude variations in APW signals and enhance model robustness to absolute signal strength, we apply random scaling factors to the signal amplitude.

We set 0.005 as the noise level, 20 points as the maximum offset, and (0.95, 1.05) as the amplitude scaling range.

### 3.4 Parameter setting

We design and validate the deep learning models in a Python environment using the Pytorch library on a computer with a 14-core CPU and 32 GB RAM. The graphics card used is an NVIDIA GeForce RTX 4070 Ti Super 16GB to accelerate model training.

The 1DCNN module consists of three convolutional blocks with kernel sizes of 7, 5, and 3 and filter counts of 32, 64, and 128, respectively. The BiLSTM module comprises two stacked blocks with 256 hidden units each. The Transformer block is composed of three transformer blocks connected in parallel, each equipped with eight attention heads. Three fully connected layers are appended at the end for classification.

We apply the cross-entropy loss function suitable for classification tasks. We set 0.001 as the initial learning rate and 32 as the batch size, train the model for 100 epochs applying the Adam optimizer, and stop training if performance does not improve after 15 epochs. To ensure the stability and generalization ability of the model, we adopt 5-fold cross-validation for data partitioning.

### 3.5 Evaluation metrics

We use accuracy rate, precision rate, recall rate, and F1 score to evaluate the model classification performance (Equations 10–13).


$$\text{Accuracy}=\frac{\mathrm{TP}+\mathrm{TN}}{\mathrm{TP}+\mathrm{TN}+\mathrm{FP}+\mathrm{FN}}$$ (10)

$$\text{Precision}=\frac{\mathrm{TP}}{\mathrm{TP}+\mathrm{FP}}$$ (11)

$$\text{Recall}=\frac{\mathrm{TP}}{\mathrm{TP}+\mathrm{FN}}$$ (12)

$$F1=2\times \frac{\text{Precision}\times \text{Recall}}{\text{Precision}+\text{Recall}}$$ (13)

TP is the number of samples that are actually hypertensive and correctly predicted as hypertensive, TN is the number of samples that are actually non-hypertensive and correctly predicted as non-hypertensive, FP is the number of samples that are actually non-hypertensive but wrongly predicted as hypertensive, and FN is the number of samples that are actually hypertensive but wrongly predicted as non-hypertensive.

## 4 Results

### 4.1 Model performance evaluation

To assess the effectiveness and computational complexity of the 1DCNN-BiLSTM-Transformer model, we compare it against five representative deep learning models commonly used for pulse wave-based hypertension classification (Tables 2, 3).

**Table 2:** Model performance comparison.

Points	Models	Accuracy (%)	Precision (%)	Recall (%)	F1 Score (%)
Right-Cun	1DCNN-RF	76.4	73.8	72.5	73.1
	LSTM-RF	79.1	76.5	75.2	75.8
	1DCNN-LSTM-RF	81.8	79.3	78.1	78.7
	STFT-CNN-SVM	80.8	77.2	76.0	76.6
	WT-CNN-SVM	81.8	79.1	77.9	78.5
	1DCNN-BiLSTM-Transformer	87.3	85.2	84.0	84.6
Right-Guan	1DCNN-RF	74.5	71.9	70.3	71.1
	LSTM-RF	77.2	74.8	73.6	74.2
	LSTM-1DCNN-RF	79.1	76.9	75.7	76.3
	STFT-CNN-SVM	78.2	75.8	74.6	75.2
	WT-CNN-SVM	79.1	76.7	75.5	76.1
	1DCNN-BiLSTM-Transformer	85.5	83.4	82.2	82.8
Right-Chi	1DCNN-RF	72.7	70.2	68.9	69.5
	LSTM-RF	75.5	73.0	71.8	72.4
	LSTM-1DCNN-RF	77.3	75.0	73.8	74.4
	STFT-CNN-SVM	76.4	73.9	72.7	73.3
	WT-CNN-SVM	77.3	75.0	73.8	74.4
	1DCNN-BiLSTM-Transformer	83.6	81.1	79.9	80.5
Left-Cun	1DCNN-RF	78.2	75.6	74.3	74.9
	LSTM-RF	80.9	78.7	77.5	78.1
	LSTM-1DCNN-RF	83.6	81.5	80.3	80.9
	STFT-CNN-SVM	81.8	79.6	78.4	79.0
	WT-CNN-SVM	83.6	81.2	80.0	80.6
	1DCNN-BiLSTM-Transformer	92.7	90.8	89.6	90.2
Left-Guan	1DCNN-RF	75.5	73.1	72.0	72.5
	LSTM-RF	78.2	76.3	75.1	75.7
	LSTM-1DCNN-RF	80.9	78.8	77.6	78.2
	STFT-CNN-SVM	79.1	76.9	75.7	76.3
	WT-CNN-SVM	80.9	78.6	77.4	78.0
	1DCNN-BiLSTM-Transformer	89.1	87.3	86.1	86.7
Left-Chi	1DCNN-RF	73.6	71.4	70.1	70.7
	LSTM-RF	76.4	74.2	73.0	73.6
	LSTM-1DCNN-RF	79.1	76.9	75.7	76.3
	STFT-CNN-SVM	77.3	75.1	73.9	74.5
	WT-CNN-SVM	79.1	76.9	75.7	76.3
	1DCNN-BiLSTM-Transformer	86.4	86.5	83.3	83.9

**Table 3:** Computational complexity comparison.

Models	Model complexity		Training time	Inference speed
	Params (M)	Size (MB)	Time per epoch (s)	Throughtput (seq/s)
1DCNN-RF	1.25	5.0	45.2	890
LSTM-RF	1.68	6.7	58.9	680
1DCNN-LSTM-RF	2.45	9.8	82.3	380
STFT-CNN-SVM	1.92	7.7	65.8	520
WT-CNN-SVM	2.18	8.7	73.5	450
1DCNN-BiLSTM-Transformer	2.36	9.4	79.8	420

In comparison with the five benchmarking models, our proposed 1DCNN-BiLSTM-Transformer model achieves the best performance across all six collection points. Notably, this superior performance is attained without incurring additional computational complexity.

### 4.2 Ablation experiments

**Figure 4:**
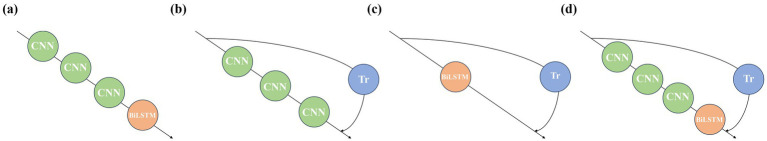
Model architectures corresponding to the ablation experiments: **(a)** Experiment 1, **(b)** Experiment 2, **(c)** Experiment 3, and **(d)** Experiment 4.

To verify the positive guiding role of each sub-module in the classification process of the 1DNN-BiLSTM-Transformer model, we conduct the ablation experiment with the APW data on the left-cun as the experimental object (Figure 4). The results are shown in Table 4.

**Table 4:** Ablation experiments results.

Experiments	Sub-Module			Evaluation metrics			
	1DCNN	BiLSTM	Transformer	Accuracy (%)	Precision (%)	Recall (%)	F1 Score (%)
1	√	√		86.2	84.1	82.3	83.2
2	√		√	88.4	86.7	85.1	85.9
3		√	√	85.9	83.5	81.8	82.6
4	√	√	√	92.7	90.8	89.6	90.2

In Experiment 1, removal of the Transformer module results in a 6.5% drop in accuracy. In Experiment 2, omitting the BiLSTM module leads to a 4.1% decline in accuracy. These findings indicate that both the Transformer and BiLSTM modules positively contribute to the model’s performance. In Experiment 3, the absence of the 1DCNN module leads to a 6.8% decrease in accuracy. Compared with Experiments 1 and 2, the decrease in accuracy in Experiment 3 is the greatest, which indicates that the local waveform features extracted by the 1DCNN module under each pressure segment are crucial for classification, and the positive guiding role of the 1DCNN module is the strongest. In Experiment 4, the original model outperforms ablation models in every metric, which confirms the synergistic effectiveness of the three modules of 1DCNN, BiLSTM, and Transformer.

## 5 Discussion

### 5.1 Comparison of classification performance under the six points

**Figure 5:**
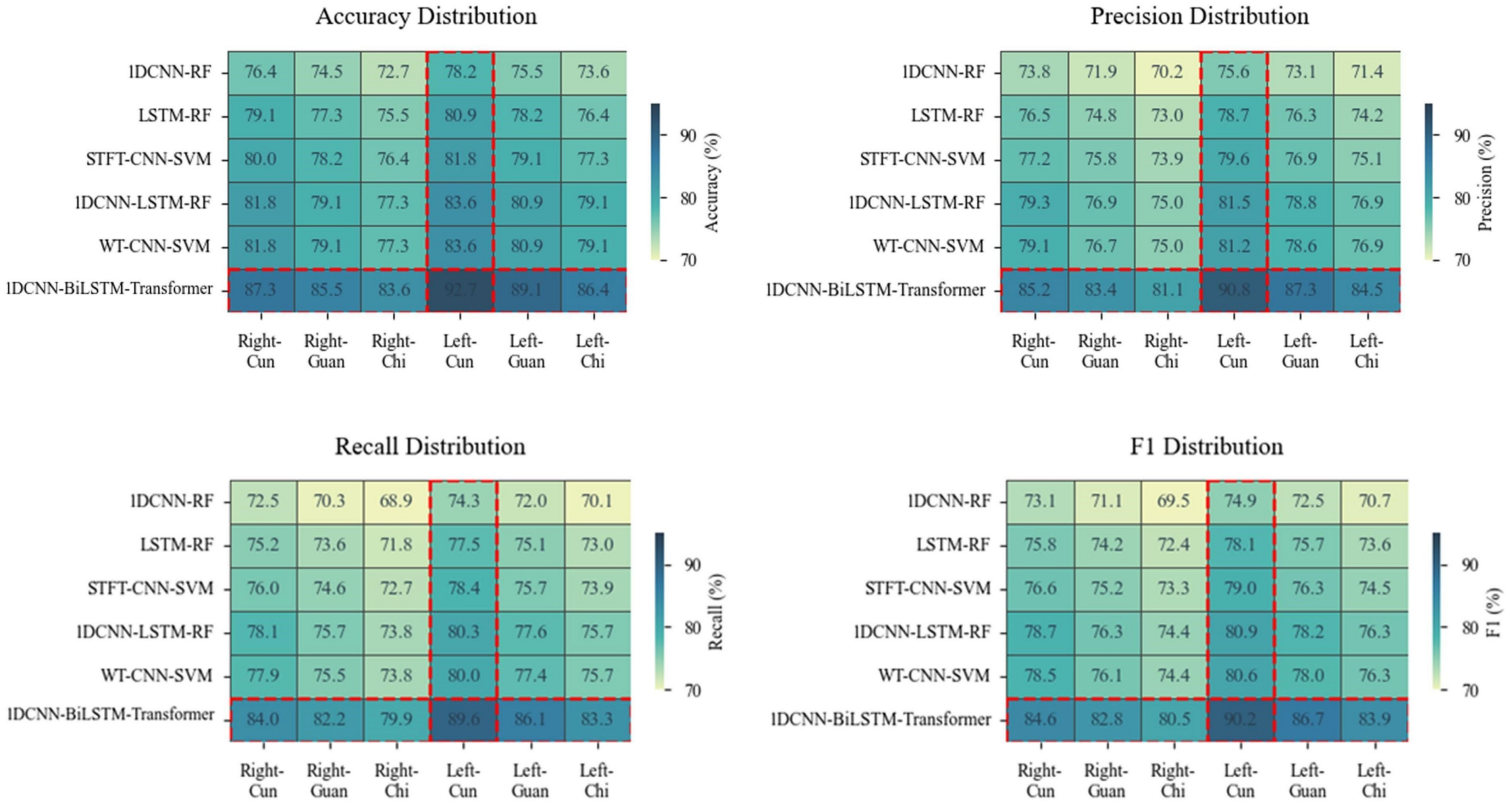
Heatmaps depicting differences in accuracy, precision, recall, and F1 score across six models at six points. A darker color indicates a higher value of the metric and a better performance.

As shown in Figure 5, the performance ranking of the six models at the six collection points from high to low is left-cun, left-guan, right-cun, left-chi, right-guan, and right-chi. The performance of the collection points on the left hand is generally greater than that on the right hand.

The left meridian belongs to Yin and governs blood, corresponds to the heart, liver, and kidneys, and directly participates in the regulation of blood circulation. Hyperactivity of liver Yang and insufficiency of kidney Yin are both core pathogenesis of hypertension (Zhang et al., 2019). The physiological characteristics of the liver storing blood and the heart governing blood vessels enable the left meridian to more sensitively reflect the hemodynamic changes of hypertension (Sun et al., 2016). The internal organ of the left-cun is the heart, and the left-cun is the closest to the aorta, which retains most of the ventricular ejection features (Wei et al., 2015). From the perspective of gut microbiota etiology, the systemic pathological changes triggered by its dysregulation—such as immune-inflammatory responses and abnormal metabolites—primarily affect the functions of organs closely associated with circulatory regulation, including the heart, liver, and kidneys. These alterations can be effectively captured by the APW signals acquired from the left meridian.

The right meridian belongs to Yang and governs qi, corresponds to the life gate of the lung and spleen, and is in charge of the ascending and descending of qi and metabolism (Zhang et al., 2019). The internal organs of the right-chi are the life gate and large intestine, and the correlation between the large intestine and blood pressure regulation is relatively weak (Zhong et al., 2019). From the perspective of gut microbiota etiology, although intestinal dysbiosis can influence systemic status through multiple pathways, the functions of the lung, spleen, and large intestine—corresponding to the right meridian—are more closely associated with the diffusion and descent of functional dynamics (qi movement) and the metabolism of water and nutrients, which only indirectly influence blood pressure. Therefore, the APW signals acquired from the right meridian are less capable of reflecting the direct vascular pathophysiological changes induced by gut microbiota dysbiosis.

### 5.2 Attention weight analysis

**Figure 6:**
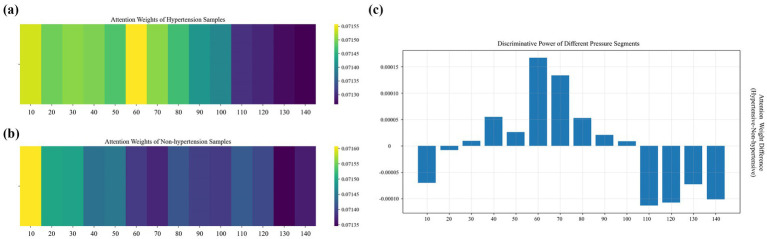
Distribution of weights across pressure segments under 14 step-pressure gradients ranging from 10 to 140 mmHg at 10 mmHg intervals: **(a)** hypertensive sample, **(b)** non-hypertensive sample, and **(c)** inter-sample differences. In **(a,b)**, a brighter color indicates a greater weight.

To explain the differences in individualized physiological and pathological characteristics in classification decisions, we randomly select a hypertensive sample and a non-hypertensive sample from the classification results and analyze the weights of APW signals under each pressure segment to results (Quan et al., 2024) (Figure 6).

In the high-pressure range (110–140 mmHg), the weights of non-hypertensive samples are significantly higher than those of hypertensive samples. The vascular elasticity of non-hypertensive patients is so strong that their vessels can buffer pressure shock through effective dilation, allowing their APW signals to maintain relatively regular morphological characteristics even in the high-pressure segments. In contrast, hypertensive patients often experience arteriosclerosis and reduced vascular wall compliance, resulting in flattened APW signals in high-pressure segments. As a result, the model assigns greater attention to the high-pressure segments of non-hypertensive samples to capture the elastic response patterns of healthy blood vessels.

In the medium- and low-pressure range (110–140 mmHg), the attention weights for hypertensive samples are significantly higher than those of non-hypertensive samples, with the differences displaying a fluctuating distribution. These segments correspond to hemodynamic processes spanning from the systolic to the mid-diastolic phase of the cardiac cycle. Due to increased vascular resistance and elevated left ventricular afterload in hypertensive patients, their APW signals in this pressure range often display abnormal features. In contrast, APW signals of non-hypertensive patients in these segments are smoother in this pressure range, and the consistency of their physiological characteristics is higher. Therefore, the model emphasizes the medium- and low-pressure segments of hypertensive samples to capture waveform distortions related to the underlying pathophysiology of hypertension.

In the extremely low-pressure range (10–20 mmHg), non-hypertensive samples receive higher attention weights than hypertensive samples. These extremely low-pressure segments correspond to the microcirculation and venous return states. In non-hypertensive individuals, higher capillary bed openness and lower peripheral blood flow resistance allow their APW signals to reflect clear microcirculatory fluctuations even at very low pressures. By contrast, due to peripheral vascular constriction and endothelial dysfunction in hypertensive patients, their APW signals are prone to baseline drift or noise interference in the extremely low-pressure segment, resulting in the masking of effective physiological information. Therefore, the model pays enhanced attention to the extremely low-pressure segments of non-hypertensive samples to capture the differentiated features of health status under microcirculation and pays reduced attention to the extremely low-pressure segments of hypertensive samples to avoid the negative impact of noise interference on the classification results.

### 5.3 Grad-CAM analysis

Grad-CAM (Gradient-weighted Class Activation Mapping) is a visualization technique used to interpret the decisions of deep learning models by computing the gradient of the target class with respect to the last convolutional layer, generating a heatmap reflecting the regions of interest of the model, with higher-weighted regions contributing more to the prediction results.

**Figure 7:**
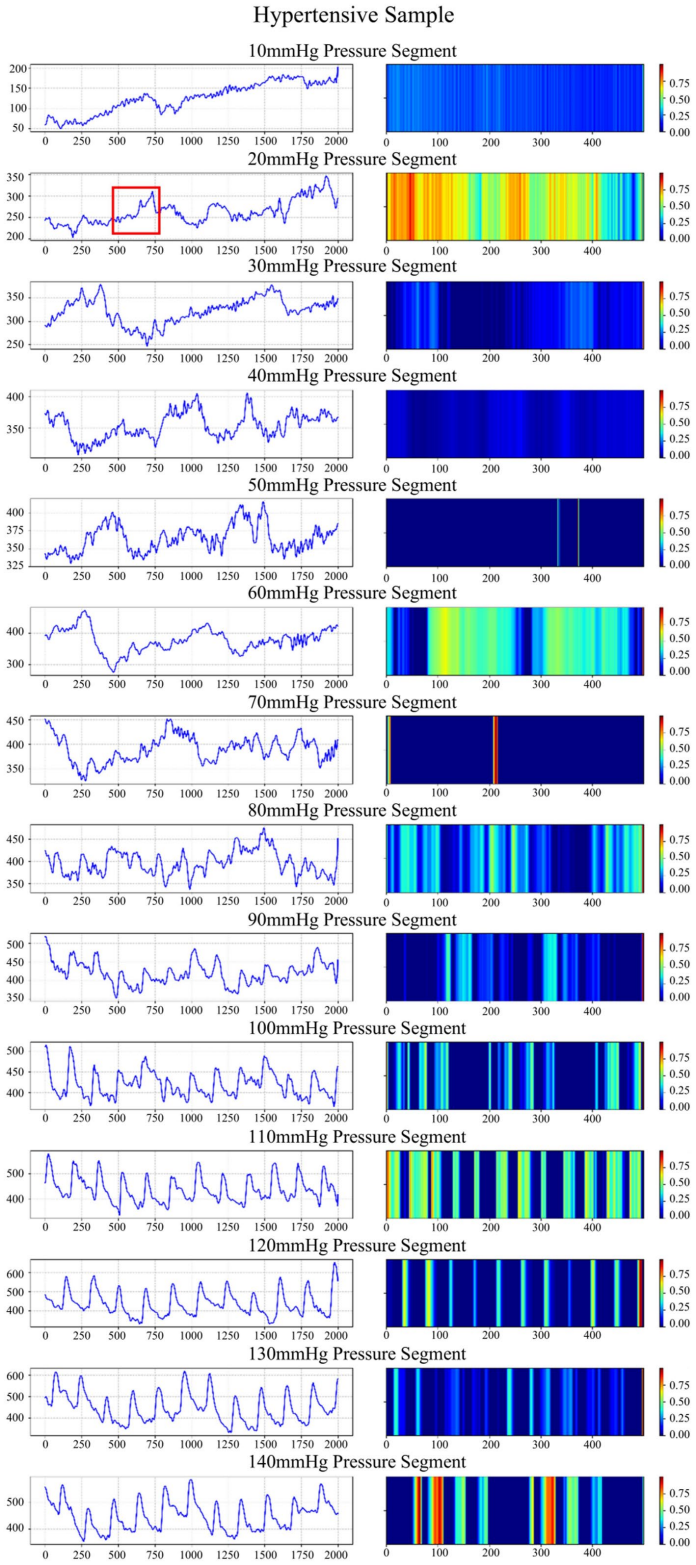
Waveforms and time-frequency heatmaps across 14 pressure segments for a hypertension sample. In the heatmaps, a brighter color indicates a greater weight. The region marked in red corresponds to the waveform with the highest activation in the heatmap.

We randomly select a hypertension sample and apply an improved Grad-CAM algorithm to generate time-frequency heatmaps under 14 pressure segments (Figure 7), along with the corresponding waveform. To validate the clinical significance of the waveform highlighted, we extract the waveform corresponding to the region with the highest activation in the heatmap and compare it with clinical waveform morphologies associated with hypertension (Nan et al., 2025; Hu et al., 2024). The results indicate that this waveform is located after the primary systolic peak and is highly consistent with the characteristic hypertensive waveform—the augmented reflection wave zone (Huang et al., 2018). The observed late-systolic peak in this region suggests early wave reflection due to increased arterial stiffness, which is a direct hemodynamic manifestation of increased left ventricular afterload. These findings support the clear clinical and pathological significance of the features identified by the model.

## 6 Conclusion

We propose a 1DCNN-BiLSTM-Transformer model for pulse wave-based hypertension classification and validate it on the APW dataset from the Population Health Data Archive (PHDA). We conduct physiologically driven interpretable analysis by evaluating model performance across different collection points, demonstrating that APW can reflect pathophysiological features linked to gut microbiota dysbiosis. We also conduct model-driven interpretable analysis employing both attention weights and Grad-CAM analysis to offer a clinical decision-making basis.

In future work, we plan to leverage APW as a non-invasive tool to explore novel therapeutic strategies for the prevention and management of hypertension. Additionally, we plan to develop an edge-deployable hypertension classification system based on our proposed model.

## Data Availability

The datasets presented in this study can be found in online repositories. The names of the repository/repositories and accession number(s) can be found in the article/supplementary material.
